# A comprehensive allele specific expression resource for the equine transcriptome

**DOI:** 10.1186/s12864-025-11240-6

**Published:** 2025-01-30

**Authors:** Harrison D. Heath, Sichong Peng, Tomasz Szmatola, Stephanie Ryan, Rebecca R. Bellone, Theodore Kalbfleisch, Jessica L. Petersen, Carrie J. Finno

**Affiliations:** 1 Department of Population Health and Reproduction, Davis School of Veterinary Medicine, University of California, Room 4206 Vet Med3A One Shields Ave, Davis, CA 95616 USA; 2 Centre of Experimental and Innovative Medicine, University of Agriculture in Kraków, Al. Mickiewicza 24/28, 30-059 Kraków, Poland; 3 Veterinary Genetics Laboratory, University of California, Davis School of Veterinary Medicine, Davis, CA 95616 USA; 4 Maxwell H. Gluck Equine Research Center, University of Kentucky, Lexington, KY 40546 USA; 5 Department of Animal Science, University of Nebraska-Lincoln, Lincoln, NE 68583 USA; 6 Present address: Eclipsebio, San Diego, CA 92121 USA

**Keywords:** Epigenetics, FAANG, Haplotype, Horse, RNA-sequencing

## Abstract

**Background:**

Allele-specific expression (ASE) analysis provides a nuanced view of cis-regulatory mechanisms affecting gene expression.

**Results:**

An equine ASE analysis was performed, using integrated Iso-seq and short-read RNA sequencing data from four healthy Thoroughbreds (2 mares and 2 stallions) across 9 tissues from the Functional Annotation of Animal Genomes (FAANG) project. Allele expression was quantified by haplotypes from long-read data, with 42,900 allele expression events compared. Within these events, 635 (1.48%) demonstrated ASE, with liver tissue containing the highest proportion. Genetic variants within ASE events were located in histone modified regions 64.2% of the time. Validation of allele-specific variants, using a set of 66 equine liver samples from multiple breeds, confirmed that 97% of variants demonstrated ASE.

**Conclusions:**

This valuable publicly accessible resource is poised to facilitate investigations into regulatory variation in equine tissues. Our results highlight the tissue-specific nature of allelic imbalance in the equine genome.

**Supplementary Information:**

The online version contains supplementary material available at 10.1186/s12864-025-11240-6.

## Background

In diploid mammalian cells, autosomal genes are usually equally expressed [1]. In some cases however, a gene can exhibit expression biased for one allele over the other [2]. This allele-specific expression (ASE) often results from cis-acting genetic variants on the same chromosome that are in proximity to or within the affected gene. Genetic variants within a gene can affect gene expression, mRNA stability, or mRNA function in different ways, leading to one allele being expressed more than the other. Various cis-acting elements have the potential to cause ASE. Although not changing the amino acid, synonymous variants could affect mRNA stability, splicing, or translational efficiency, potentially causing ASE [3]. Missense variants can affect the function of the RNA, possibly leading to a difference in expression between alleles [3]. Variants in the 3' untranslated region (UTR) can influence mRNA stability, localization, and translation, all of which can contribute to ASE. Variants identified in the 5' UTR could influence ASE by affecting the initiation of translation and the stability of mRNA thus altering the amount of protein produced from each allele [4]. Lastly, variants in splice regions can impact allele expression by altering splicing efficiency, exon skipping, creation or loss of splice sites, or affecting regulatory protein binding, all potentially influencing mRNA function and expression [4].

ASE may also arise from trans effects, or genetic influences on the other chromosome of an affected gene or elsewhere in the genome [5, 6]. In addition to cis and trans effects, epigenetic factors such as DNA methylation or chromatin structure also have the potential to significantly impact gene expression between alleles [1, 6]. Interestingly, ASE predominantly manifests as tissue-specific phenomena, with loci displaying distinct expression patterns across different tissues [2, 5]. Therefore, ASE analysis can provide a way to inspect gene regulation patterns and their broader impact on biological pathways in specific tissues.

Advances in next-generation sequencing technologies, particularly RNA-sequencing (RNA-Seq), have revolutionized our ability to analyze gene expression and genetic variation. Furthermore, long-read RNA-Seq allows for the straightforward identification of variants that are inherited together as haplotypes from full-length transcripts [7, 8]. The loci of these haplotypes can then be overlaid with short-read RNA-Seq reads. This integration provides the read counts of nucleotides existing at each locus which can then be used to quantify the expression level of each allele for each gene that contains heterozygous loci. Finally, expression levels of each allele can be compared against one another to identify an allelic imbalance.

The study of ASE in horses is enabled by the availability of a high-quality reference genome [9] and long- and short-read sequencing technologies, contributing to a more comprehensive transcriptome annotation [10, 11]. While prior studies on ASE in horses were focused on paternal/maternal imprinting in early development, this research distinguishes itself by examining ASE in fully developed mares and stallions [12–14]. In this study, we performed an ASE analysis of the equine transcriptome using a combinatorial Iso-seq and RNA sequencing approach. Our primary goal of this research was to contribute to the Functional Annotation of the Animal Genome (FAANG) project, a large-scale collaborative effort aimed to identify all functional elements for animals [15]. To accomplish this, we provide a comprehensive resource of allelic expression detected from linked heterozygous loci. We have designed this publicly accessible database to enable future research into important regulatory variants that may impact equine health and disease at the molecular level. By extending the examination of ASE to the equine species using this method, we can further understand the uses of emerging next-generation sequencing technologies and the mechanisms underlying gene regulation in the equine genome.

## Methods

### Generation of data

Data from nine tissues from prior FAANG analyses were selected for ASE analyses. These tissues included the lamina, liver, left lung, left ventricle of the heart, longissimus muscle, skin, parietal cortex, testes, and ovary from 4 healthy horses of the Thoroughbred breed (2 mares and 2 stallions) [16, 17]. This selection aimed to encompass a broad spectrum of biological functions and, consequently, varied gene expression.

The RNA isolation for Iso-seq was performed separately from the same tissues as the RNA utilized for mRNA-Seq, using an identical protocol [11]. For Iso-seq, we selected the highest quality RNA per sex for each tissue (except for parietal cortex and sex-specific tissues). Since parietal cortex was the pilot tissue, long-read RNA sequencing was performed on all four horses. All selected RNA samples had integrity (RIN) values greater than or equal to 7. Selected tissues were processed for Iso-seq in a single batch. The cDNA libraries were developed and sequenced at the UC Berkeley QB3 Genomics core facility. Two randomly selected libraries were combined and then sequenced together on a single SMRT cell of the PacBio Sequel II system, as previously described [10, 11].

ChIP-seq data for the histone modifications evaluated in this study were sourced and analyzed from prior publications that examined the same eight tissues from the same horses used in this study [11, 18].

### Identifying haplotypes

The overall workflow for identifying ASE events is outlined in Supplementary Fig. 1. Key to our approach was the use of Iso-seq data to extrapolate haplotypes for each horse individually, utilizing the isophase Cupcake software v29.0 [19]. First, circular consensus sequencing reads were generated by the CCS algorithm. Reads with a quality score below Q20 were excluded. Next, concatemers and reads with poly-A tails less than 20 base pairs were removed. Following this, the software's isoform function was used to identify variations between otherwise identical full-length reads. Lastly, the high-quality long reads were then aligned to Equcab.3.0 [9] to pinpoint the loci of heterozygous SNPs.

### Integration of short-read RNA library

To prepare short-read RNA-Seq data for analysis, sequences were trimmed to remove adapters low-quality read, and PCR duplicates using trim-galore v0.6.10 [20] and Cutadapt v4.7 [21]. Read qualities were inspected using fastQC v0.11.7 [22] and multiQC v1.16 [23]. Reads shorter than 50 bp or with a quality score below 30 were filtered out. The heterozygous SNP loci identified from high-quality long-read sequencing were then overlaid with these short-reads using SAMtools mpileup from SAMtools 1.18 [24]. We ensured that both the short-read and long-read RNA-Seq analyses were based on the same tissues and samples.

### Quantify expression for heterozygous loci

After overlaying reads found at the heterozygous loci, we quantified the expressed nucleotides at these positions. This was accomplished using a custom Python tool [28]. The fundamental use behind this tool is to tally the occurrences of each nucleotide variant at a given locus from the output of the SAMtools program. For example, if a particular position has both 'A' and 'G' variants, the tool calculates the frequencies of 'A' and 'G' in the reads. This process is repeated across all identified heterozygous sites in the genome.

### Quantify allele expression per haplotype

The expression values from these heterozygous positions were then aggregated to quantify expression values representing each allele. Expression values of each nucleotide at each heterozygous loci were summed according to their respective haplotype sequence. When a certain haplotype sequence is expressed more frequently compared to its counterpart, it is evidence that this particular allele is being expressed at a higher level.

The distribution of read counts across all samples is provided in *supplementary materials* (Supplementary Fig. 2).

### Identify significant allele specific expression

After deriving allele expression, we then pinpointed ASE using filtering and statistical techniques outlined below. First, we excluded cases where neither of the allele expression values being compared met or exceeded a threshold of 10. We then assessed ASE by examining the differential expression between haplotypes. To do so, we applied a log transformation to calculate allele expression fold change (aeFC) between both allele expression values. The equation for aeFC is shown below:

$$allele\;exp.\;fold\;change=log2\;\left(allele\;1\;exp.value\right)-log2\;\left(allele\;2\;exp.value\right)$$

The distribution of aeFC across all samples is provided in supplementary materials (Supplementary Fig. 3).

To supplement aeFC, we used an additional statistical test to examine disparities between calculated expression values. Operating under the null hypothesis that allele expression values for any gene should be roughly equivalent between alleles, we calculated p-values using a binomial test. Considering the count-based nature of our data, we employed the Benjamini–Hochberg procedure to manage the expected false discovery rate, yielding adjusted p-values.

For the final stage of significant ASE identification, we established stringent criteria to distinguish instances of significant allele expression imbalance: the aeFC between two alleles had to be at least an absolute value of 2, the adjusted p-value needed to be ≤ 0.05, and at least one of the calculated allele expression values be ≥ 5.

### Incorporating histone modification data

ChIP-seq data were integrated by defining regions spanning from the first to the last heterozygous position within our haplotype transcripts. Leveraging this defined region, we executed computational assessments to ascertain any overlaps with key chromatin peaks, specifically H3K27me3, H3K27ac, H3K4me1, and H3K4me3, on a tissue and sample basis.

### Identify genes with ASE

We identified the gene names of loci exhibiting ASE using Ensembl’s Variant Effect Predictor (VEP) v10 [25]. For each heterozygous locus differentiating isoforms, VEP returned the associated gene. VEP may provide multiple annotations for a single variant, therefore, variants predicted as intronic or intergenic were filtered out to only annotate variants strictly from RNA-Seq. Variants annotated exclusively as intergenic or intronic were excluded from downstream analyses. To view the data frame of variants that were filtered out see Additional File 1.

### Validation of ASE events

Our validation set consisted of short-read RNA-seq data from 66 liver samples, 41 males and 25 females, with no evidence of liver disease. These samples were comprised of 12 breeds including; 27 Quarter Horses, 17 Warmbloods, 8 Thoroughbreds, 3 Andalusians, 3 Arabians, 2 Lusitanos, 1 Percheron, 1 Shire, 1 Ponys of the Americas, 1 Friesian, 1 Mustang, and 1 Gypsy Vanner. The average age of this cohort was 3.49 years, and ranged from 1 month to 8 years of age. RNA-seq was performed with Illumina polyA-selection with a read length of 2 × 150 bp. Adapter trimming, poly-A trimming, N trimming, quality trimming, length filtering, and removal of PCR duplicates were conducted using HTStream, version 1.3.3 [26]. Reads were aligned to Equcab.3.0 [9] using STAR, (v2.7.10b) [27]. Expression counts at identified ASE loci in liver were generated using SAMtools mpileup from SAMtools 1.18 [24]. Loci that were not heterozygous were filtered out. We used the same significance criteria as our original cohort—aeFC ≥ 2 and adjusted p-value ≤ 0.05. To view the data frame of expression values in these loci see Additional File 2.

### Allele specific differentially expressed gene enrichment analysis

Following the validation of ASE events, we investigated their broader biological implications through gene enrichment analysis (GEA) using KOBAS [28] (KEGG Orthology-Based Annotation System). Our method involved a deliberate combination of allele specifically expressed genes (ASDEGs) from various samples on a tissue-specific basis. Inputting these lists of genes into KOBAS for each tissue type we could identify pathways with a significant proportion of ASDEGs. P-values were supplied by KOBAS for each impacted pathway, and significant pathways were identified as having a p-value ≤ 0.05.

Dataframe management and statistical analyses were performed using scipy [29], numpy [30], and pandas [31]. Data visualization was carried out with matplotlib [32] and seaborn [33].

## Results

### Allele specific expression in the horse genome

**Fig. 1:**
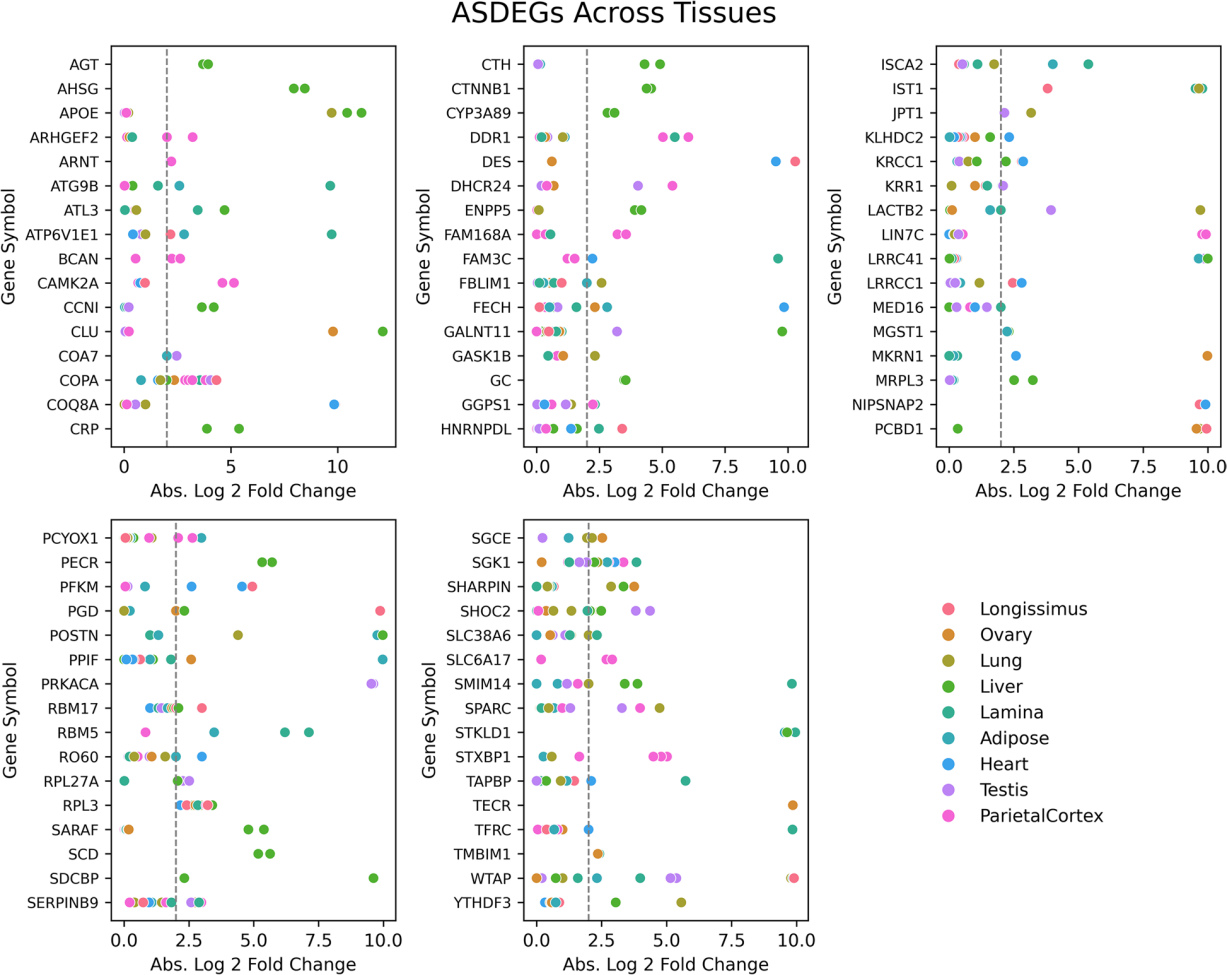
ASDEGs Comparisons Across Tissues. The scatter plots display the absolute log2-fold change in allele expression for identified ASDEGs across various tissues. Each gene featured has at least 2 identified ASE events across all horses and tissues. ASDEGs are graphed in alphabetical order. Each dot represents the expression fold change for a gene in a specific tissue, plotted against the gene symbol on the x-axis and the absolute log2-fold change on the y-axis. The dotted line indicates the significance threshold of a twofold change. The color coding corresponds to different tissues, as indicated in the legend

Recent data from the equine functional annotation of animal genomes (FAANG) initiative was leveraged for this analysis [10]. Haplotypes for each horse/tissue sample were identified from Iso-seq data. Across all samples, we identified 87,174 heterozygous loci. Using these loci to differentiate alleles, and subsequently quantifying the nucleotide reads at these positions using associated short-read RNA data from the same horse/tissue sample, 42,900 allele expression events were compared. After filtering and performing statistical analyses described in *Methods*, we compiled this data into an allele expression resource (see Additional File 3). Using this resource, we identified 635 (1.48%) of allele expression events as having demonstrated ASE. ASE was detected in all analyzed tissues, with the liver containing the highest proportion of ASE occurrences. (Table 1) Among the genes showing evidence of ASE, referred to here as allele specific differentially expressed genes (ASDEGs), 80 exhibited ASE in more than one tissue or sample (Fig. 1).

**Table 1:** Distribution of analyzed genes across tissues. An overview of the alleles examined across multiple tissue types. The "Alleles Comparisons" column enumerates the number of allele comparisons within each specific tissue type (2 alleles for each comparison). The "Significant Allele Imbalance" column identifies the subset of these alleles that exhibited notable expression differences from the expected equilibrium in our study

Tissue	Allele Comparisons	ASE	%
Liver	3404	135	3.97
Heart	3031	62	2.05
Longissimus	3100	58	1.87
Adipose	4855	89	1.83
Lamina	4413	64	1.45
Ovary	3782	51	1.35
Lung	4110	55	1.34
Pareital Cortex	5203	61	1.17
Testis	5253	60	1.14

### Investigating heterozygous loci

**Fig. 2:**
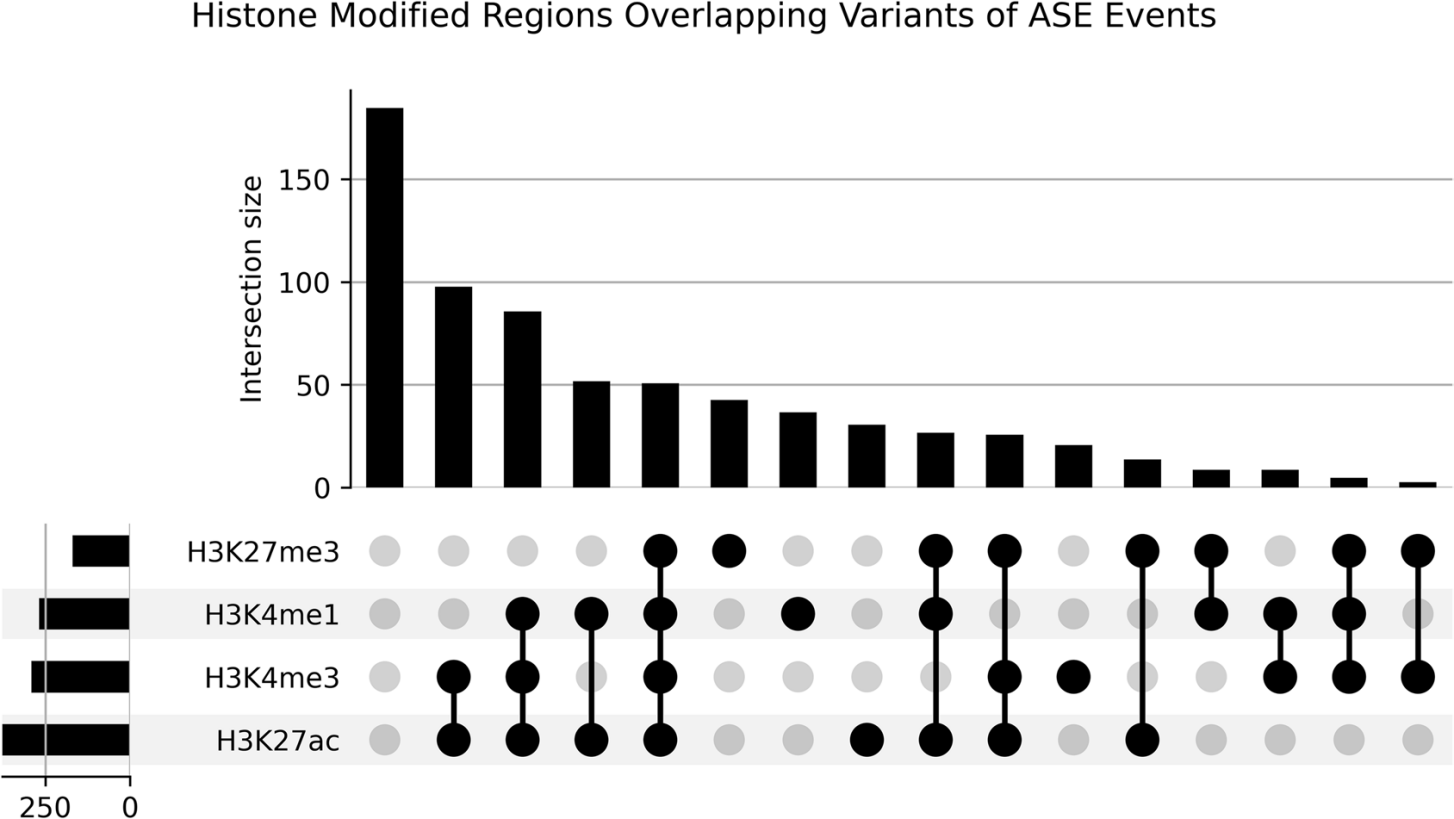
Histone Modified Regions Overlapping Variants of ASE Events. Upset plot representing the distribution and overlap of histone modifications across heterozygous loci associated with allele-specific differentially expressed genes (ASDEGs). Each circle corresponds to a specific histone modification as indicated by the legend (H3K27ac, H3K4me1, H3K27me3, H3K4me3). Circles, and their respective frequency bars, denote the count of SNP regions that exhibit the corresponding histone modifications

A total of 774 heterozygous loci were identified in ASE events. Variant effects were predicted for these loci, and approximately 43% were located within 3' untranslated regions (Table 2). A total of 497 (64.2%) of the identified variants in ASE events fell within histone modified regions. The majority of ASE events were associatedwith H3K27ac peaks (*n* = 377, 55.3%), followed by H3K4me3 peaks (*n* = 293, 43.0%), H3K4me1 peaks (*n* = 268, 39.4%), and H3K27me3 peaks (*n* = 170, 24.9%). From the 497 ASE events identified as having SNPs associated with histone modification regions, 369 (74.2%) showed overlap of multiple histone marks. The three most common overlapping histone modification regions with identified variants were H3K4me3 and H3K27ac, H3K27ac and H3K4me1, and H3K27ac with H3K4me1 and H3K4me3 (Fig. 2).

**Table 2:** Variant types detected in ASE events variant types identified in allele-specific expression events within the study's sample set. Variant predictions were made using VEP [25]

Variant Types in ASE Events	Count	%
3 prime UTR variant	335	43.28
missense variant	135	17.44
synonymous variant	134	17.31
5 prime UTR variant	131	16.93
non coding transcript exon variant	9	1.16
splice region variant & splice polypyrimidine tract variant & intron variant	6	0.78
splice region variant & synonymous variant	4	0.52
missense variant & splice region variant	4	0.52
splice donor region variant & intron variant	4	0.52
stop lost	4	0.52
stop gained	3	0.39
splice region variant & 5 prime UTR variant	2	0.26
splice polypyrimidine tract variant & intron variant	1	0.13
splice region variant & intron variant	1	0.13
missense variant & stop retained variant	1	0.13

### Differentially expressed gene enrichment analysis

**Fig. 3:**
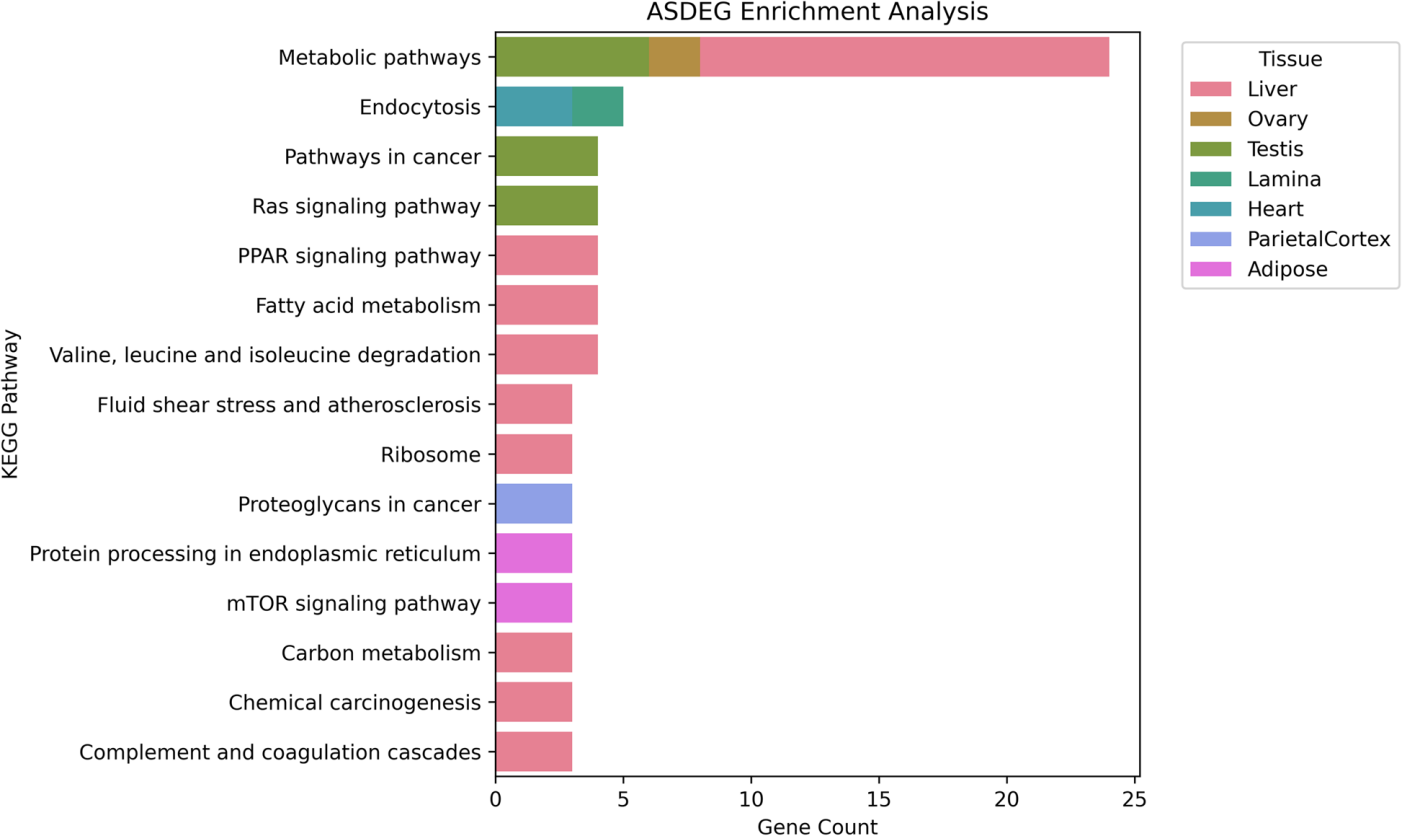
ASDEG enrichment analysis. Bar chart depicting the number of ASDEGs (Allele Specific Differentially Expressed Genes) present within significantly enriched pathways identified in each tissue type from KOBAS enrichment analysis. Tissues analyzed include Liver, Ovary, Testis, Lamina, Heart, Parietal Cortex, and Adipose

We identified 168 KEGG pathways containing a significant proportion of ASDEGs, including metabolic pathways, endocytosis, and the Ras signaling pathway. In our study, the liver contained the greatest number of pathways significantly impacted by ASE (Fig. 3).

### Validation of allele specific variants

**Fig. 4:**
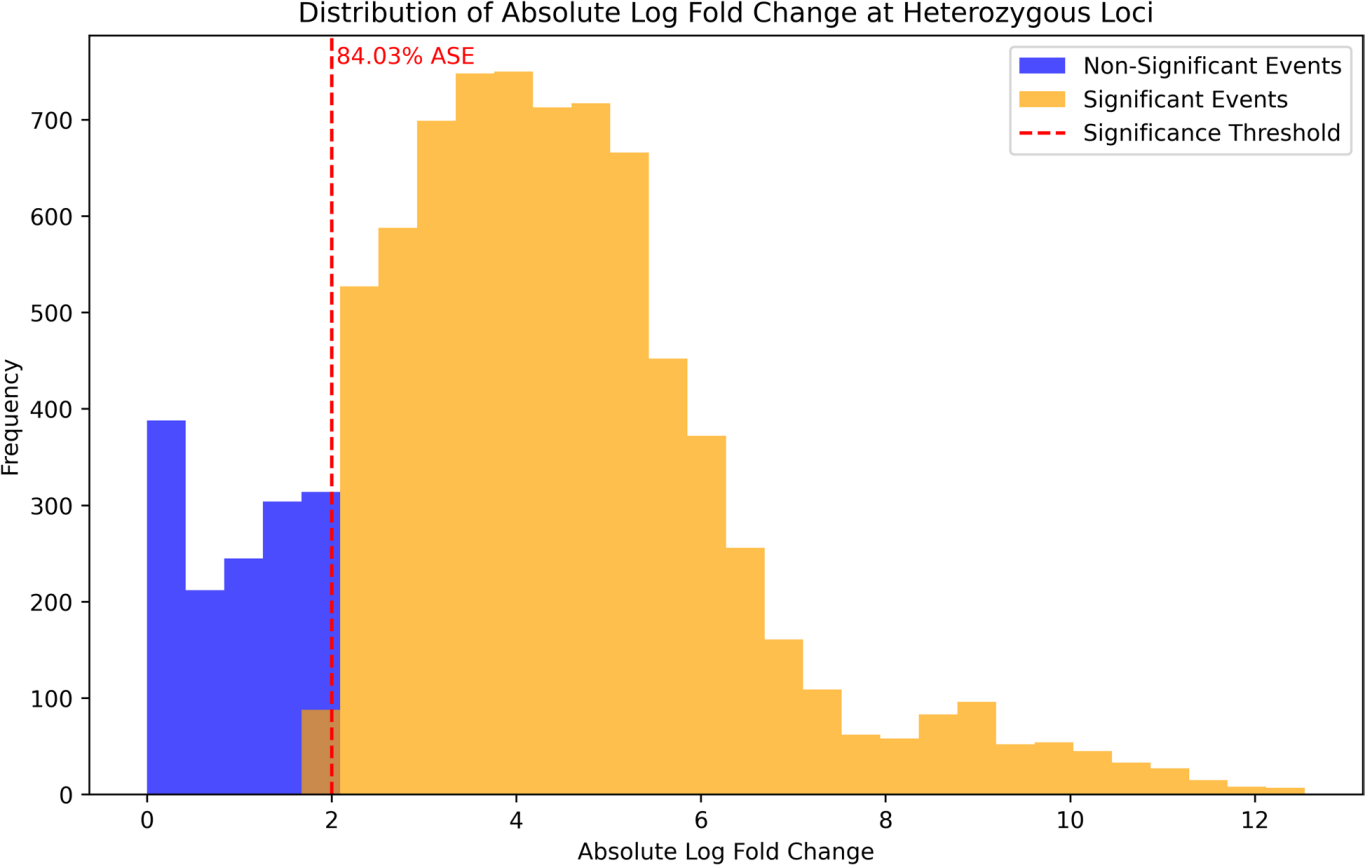
Validation of ASE in Heterozygous Loci in Liver Tissue from Independent Dataset. Distribution of absolute log-fold changes at heterozygous loci identified within allele-specific expression (ASE) events in liver tissue, and their overlay with a validation set of short-read RNA-seq data. Two categories are presented: non-significant events (blue) and significant events (orange) that exhibit ASE. The dashed red line indicates the significance threshold, with the loci to the right deemed to show significant ASE

To validate our putatively identified ASE loci in liver tissue, we examined the loci in a larger dataset of liver tissue, consisting of 66 samples from horses of various breeds. A significant proportion of the examined loci also exhibited ASE in this validation cohort. All 155 heterozygous loci identified in liver tissue from our original FAANG horses were tested in the validation set, resulting in 8,849 heterozygous loci expression comparisons. Specifically 7,436 (84%) of the comparisons made across all *n* = 66 samples in our validation set were confirmed to show ASE, with 7019 (94.4%) of these comparisons in the same direction (i.e. allele A demonstrates higher expression and allele B demonstrates lower expression: Fig. 4). Among the 155 ASE loci tested, 96.7% showed ASE in at least one of the samples used in our validation set, with 85 (54.8%) showing ASE in at least 90% of the *n* = 66 samples tested (Supplementary Fig. 4). ANOVA results indicated a statistically significant difference in AEFC between breeds (F(11, 283,416) = 104.27, p < 0.001). However, the effect size, as measured by eta-squared, was extremely small (η^2^ = 0.004), suggesting that only 0.4% of the total variance in ASE can be attributed to breed. While a breed effect is present in a statistical sense, it is unlikely to be meaningful in this biological context (Supplementary Fig. 5). Pictures of validated ASE loci displayed in Integrative Genomics Viewer [34] can be viewed in Supplemental Figs. 6–8.

## Discussion

### ASE analysis

The foundation of our ASE analysis was the identification and assignment of heterozygous loci. The incorporation of full length transcripts from Iso-seq from the equine FAANG initiative was instrumental in this aspect. Short-read RNA-Seq’s segmented view of genetic sequences presents challenges in congruent sequence construction [1]. On the other hand, Iso-seq’s unfragmented view of the transcriptome facilitates a more robust identification of haplotypes [7, 8]. This methodology surpasses evaluations based solely on expression values of individual heterozygous variants, ensuring a more thorough and accurate assessment [6–8].

To minimize the inclusion of artifacts or minor variations that may not reflect true differential expression, we excluded ASE events with variants that were only identified as within intergenic or intronic regions. Given the nature of RNA-Seq data, this filtering ensures that the analysis prioritizes variants that are most pertinent to the corresponding genes. This supplementary data could possibly be used to extend currently annotated transcribed regions, since this data was generated strictly from high quality RNA-Seq. Additionally, we excluded cases where neither of the allele expression values being compared met or exceeded a threshold of 10 reads. Lastly, sequencing data from sex chromosomes was also filtered out because of this study’s focus on autosomal genes. These filtered out events are still available for analysis in supplementary materials.

### *Tissue regulation *via* ASE*

One of the main goals of this study was to identify tissue specific ASE. We discovered genes demonstrating ASE in one tissue, while exhibiting equal expression across alleles in other tissues. This finding suggests that the regulatory mechanisms contributing to these ASDEGs are unique to the particular tissues where they were found [1, 2]. Such specificity could be due to tissue-specific promoters, enhancers, or other regulatory elements that influence gene expression differently in each tissue type [1, 3]. Apolipoprotein E gene, *APOE*, was among these genes and demonstrated ASE in two out of two liver tissues (one from each sex) used in this study, while having equal allele expression in testis, parietal cortex, and lung tissues. Notably, all APOE ASE events favored the allele with the same missense variant at locus chr10:15714449. Another gene in this study, *SLC6A17,* is a part of the SLC6 family of transporters. This gene exhibited ASE in 2 out of 4 parietal cortex tissues in this study. Interestingly, both instances of *SLC6A17* ASE were observed in parietal cortex tissue of mares, whereas stallions had a bi-alleleic expression for *SLC6A17* in parietal cortex tissues*.* Both ASE events involving *SLC6A17* favored the allele with the same 3 prime UTR variant at locus chr5:54600372. This example alludes to the use of our resource to compare ASE across sexes, helping to identify putatively sex-specific tissue regulation. Lastly, the transmembrane glycoprotein gene *ENPP5* demonstrated evidence of ASE in 2 out of 2 samples of liver tissue used in this study, while having equal expression across alleles in parietal cortex and lung tissues. Both instances of ASE involving *ENPP5* involved favoring alleles with 3 prime UTR variants at chr20:45654742.

In addition to tissue-specific ASE, we discovered ASE events that were common among multiple tissues, suggesting more broadly used regulation mechanisms. Allele specific differentially expressed genes (ASDEGs), demonstrating ASE in more than 4 out of 9 tissues from this study, included tubulin gene *UBA1* (9 tissues), L ribosomal protein *RPL3* (8 tissues), serine/threonine protein kinase gene *SGK1* (6 tissues), *WT1*-associating gene *WTAP* (5 tissues), Coat Complex Subunit Alpha gene *COPA* (5 tissues), and lysine-rich coiled-coil protein *KRCC1* (5 tissues).

### Histone modifications

ChIP-seq data from the same FAANG horse/tissue samples was integrated to provide a broader evaluation of allele-specific expression in the context of annotated histone modifications [21, 35]. This provides an improved perspective on the epigenetic acting influences in allele-specific gene expression, highlighting the complex interplay between genetic variations and epigenetic regulation. Variants within H3K27ac peaks, commonly found at active transcription start sites (TSS), highlight the potential relationship between coding changes within TSS and allelic expression [35]. Additionally, intersection of ASE events with H3K4me1 peaks, often associated with enhancer regions, suggests that variants within these regions could lead to enhanced expression relative to the other allele [35]. Variants were also identified within H3K4me3 peaks, which are commonly identified near promoters of actively transcribed genes. This suggests that a particular promoter sequence may be favored for transcription [35]. Similarly, the repressive mark, H3K27me3, further emphasizes the possible interplay between allele imbalance and the epigenetic landscape [35]. The co-localization of ASE with active marks such as H3K27ac and H3K4me1, often found at transcription start sites and enhancer regions, respectively, suggests that these epigenetically active domains may predispose certain alleles for increased expression by facilitating a more accessible chromatin state [35]. Simultaneously, the intersection with H3K4me3, associated with promoters of actively transcribed genes, and H3K27me3, a mark of transcriptional repression, indicates a nuanced regulatory landscape where alleles may be differentially expressed due to the combinatorial effects of epigenetic modifications [35]. These overlapping epigenetic regions may serve as hotspots for ASE, where the orchestration of gene activation and silencing is fine-tuned by histone marks and coding variants. However, it is important to note that not all impactful epigenetic markers can be found in transcribed regions. Interestingly, a large portion (~ 30%) of heterozygous loci within ASE events were not found to be within this histone modified regions. This could mean that the nucleotide variation across alleles may be directly responsible for the significant expression disparity.

### Validation

To further validate our findings, we employed short-read RNA sequencing data from 66 additional equine liver samples across various breeds of horses. Liver tissue was chosen for validation due to its high frequency of ASE events in the original cohort (Table 1). This robust validation approach confirmed the reliability and reproducibility of our ASE observations across breeds and ages of horses, reinforcing the utility of our integrated Iso-seq and short-read RNA-Seq methodologies for uncovering the complexities of gene expression regulation and the prevalence of ASE in the equine genome.

### Limitations & future direction

This study represents the first ASE analysis of its kind for the horse, however there are a few limitations to consider. First, our FAANG sample size was relatively small, and we did not analyze all tissues, which may affect the generalizability of our findings. Additionally, the absence of parental sequencing prevented us from determining the origin of heterozygous loci. It is also important to note that this combinatorial RNA-Seq method simply will not detect all instances of ASE [36]. Our reliance on heterozygous gene expression markers reduces the total number of ASE events we could identify in the study since some locations in the genome may be homozygous yet still exhibit ASE due to epigenetic factors alone, such as parental imprinting [36]. Studies utilizing both RNA-Seq and whole genome sequencing have estimated the percent of genes exhibiting ASE from 1 to 20%, depending on the strength of statistical filtering [1, 36, 37]. In this study, we used high filtering standards to declare a significant allele imbalance, and identified ASE in 1.48% of alleles compared. Despite these limitations, our method provides a valuable tool to inspect linked variants and their putatively high impact on gene expression. Future research will extend this approach to a larger set of samples and tissues. This ASE database may also be used to compare allele expression in horses with other farm animals, contributing to a broader understanding of species-specific regulatory mechanisms and evolutionary divergence in allele-specific expression across different livestock species. For example, a cross-examination of our ASE database with genes validated to show ASE in cattle [38, 39] found that genes such as *SGCE*, *MEST*, and *IGF2R* exhibited ASE in both horses and cattle. Furthermore, the identified heterozygous loci used in this analysis could be overlaid with short-read RNA-Seq from horses in different developmental stages. This may demonstrate that specific allele sequences are pertinent to development and become otherwise unneeded as the horse reaches maturity. This integration will lay the foundation for a deeper understanding of the intricate relationship between imbalances in allele expression and their role within the equine genome.

## Conclusion

This study introduces the first multi-tissue analysis of ASE specific to the equine genome, spanning 4 individual Thoroughbred horses and 9 diverse tissues, with validation of liver ASE across multiple horse breeds. Here, we provide an allele expression resource for the equine community to advance future gene regulation investigations. This resource was designed to easily allow researchers to investigate genes of interest, and set their own filtering criteria to detect allelic imbalance. Additionally, this main database was divided into tissue specific tracts, thereby allowing for tissue-specific allele expression analysis. Using this resource, we inspected the heterozygous loci across alleles, potentially responsible for significant regulation of gene expression, identified pathways containing a significant proportion of these regulatory events, and pinpointed ASE patterns on a tissue-wide scale. As a result, we have demonstrated the potential that this method provides to enrich our understanding of the intricacies of equine genetic regulation by identifying notable loci variations that putatively have a significant impact on gene expression.

## Supplementary Information

Additional file 1. Filtered Out Loci : Data frame of heterozygous loci that were filtered out from downstream analyses due to their intergenic prediction from VEP.

Additional file 2. Validation Cohort Data Frame : Data frame of allele expression values from the heterozygous loci used in our validation cohort.

Additional file 3. Allele Expression Resource : Master data frame with all of the heterozygous loci analyzed in this experiment.

Additional file 4: Supplementary Figure 1. Depiction of overall pipeline for ASE identification.

Additional file 5: Supplementary Figure 2. Distribution of read counts for heterozygous loci used in this study.

Additional file 6: Supplementary Figure 3. Distribution of aeFC for all allele comparisons in this study.

Additional file 7: Supplementary Figure 4. Frequency of ASE comparisons verified in our validation set.

Additional file 8: Supplementary Figure 5. Box and Violin Plots of the log fold change distribution of identified ASE loci across all breeds in our validation cohort. QH = Quarter Horse.

Additional file 9: Supplementary Figure 6. Pictures of a few validated ASE loci in Integrative; gene AHSG locus 3:27,077,158 (6), gene APOE locus 3:15,714,449 (7), gene CLU locus 3:56,553,608 (8).

Additional file 10: Supplementary Figure 7. Pictures of a few validated ASE loci in Integrative; gene AHSG locus 3:27,077,158 (6), gene APOE locus 3:15,714,449 (7), gene CLU locus 3:56,553,608 (8).

Additional file 11: Supplementary Figure 8. Pictures of a few validated ASE loci in Integrative; gene AHSG locus 3:27,077,158 (6), gene APOE locus 3:15,714,449 (7), gene CLU locus 3:56,553,608 (8).

## Data Availability

The master and tissue separated allele expression data frames generated and analyzed in this study are available as Supplementary Files 3-10. The short-read RNA sequencing analyzed in this study is available in the ENA and SRA repositories under the accession number PRJEB26787 (female tissues - https://www.ebi.ac.uk/ena/browser/view/PRJEB26787) and PRJEB53382 (male tissues - https://www.ebi.ac.uk/ena/browser/view/PRJEB53382). The Iso-seq data analyzed in this study is available in the ENA and SRA repositories under the accession number PRJEB53020. (https://www.ebi.ac.uk/ena/browser/view/PRJEB53020). The short-read RNA sequencing from our validation set used in this study is available in the SRA repository under the accession number SUB14222280. Histone ChIP-seq analyzed in this study is available via Kingsley et al. (https://doi.org/10.3390/genes11010003) and Barber et al (thesis; https://digitalcommons.unl.edu/animalscidiss/233/).

## Abbreviations

aeFC: Allelic expression fold change
ASE: Allele specific expression
ASDEG: Allele specific differentially expressed gene
Iso-Seq: Isoform Sequencing
RNA-Seq: RNA-sequencing
SNP: Single nucleotide polymorphism
GEA: Gene enrichment analysis
