# Supplementary material for: A comprehensive allele specific expression resource for the equine transcriptome
Source: BMC Genomics. 2025 Jan 30;26:88. doi: 10.1186/s12864-025-11240-6 (PMC11780778; doi:10.1186/s12864-025-11240-6)
Supplement: Supplementary file 4 — Additional file 4: Supplementary Figure 1. Depiction of overall pipeline for ASE identification. [file 12864_2025_11240_MOESM4_ESM.pdf]

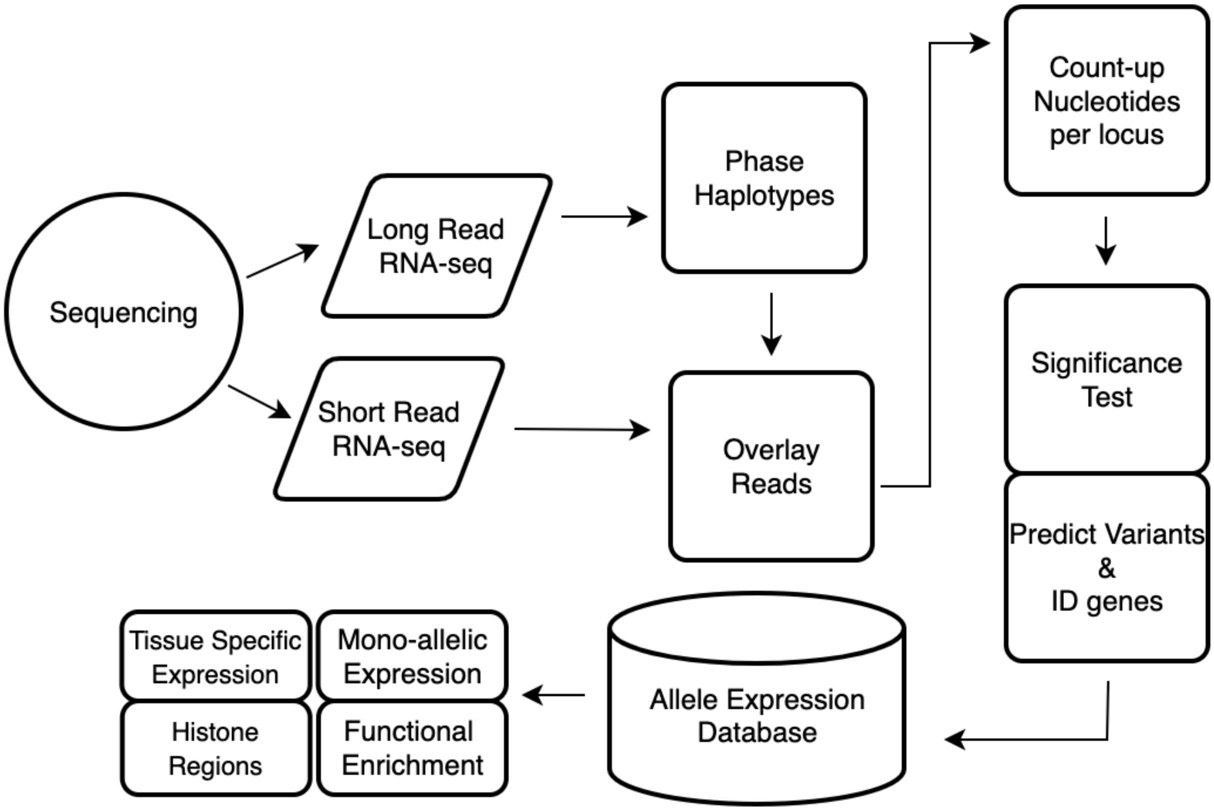

Supplementary Figure 1 - Depiction of Pipeline Used for ASE Identification : Flow chart summarizing the methods used in this study to identify and analyze the allele specifically expressed haplotypes
