# Supplementary material for: A comprehensive allele specific expression resource for the equine transcriptome
Source: BMC Genomics. 2025 Jan 30;26:88. doi: 10.1186/s12864-025-11240-6 (PMC11780778; doi:10.1186/s12864-025-11240-6)
Supplement: Supplementary file 5 — Additional file 5: Supplementary Figure 2. Distribution of read counts for heterozygous loci used in this study. [file 12864_2025_11240_MOESM5_ESM.pdf]

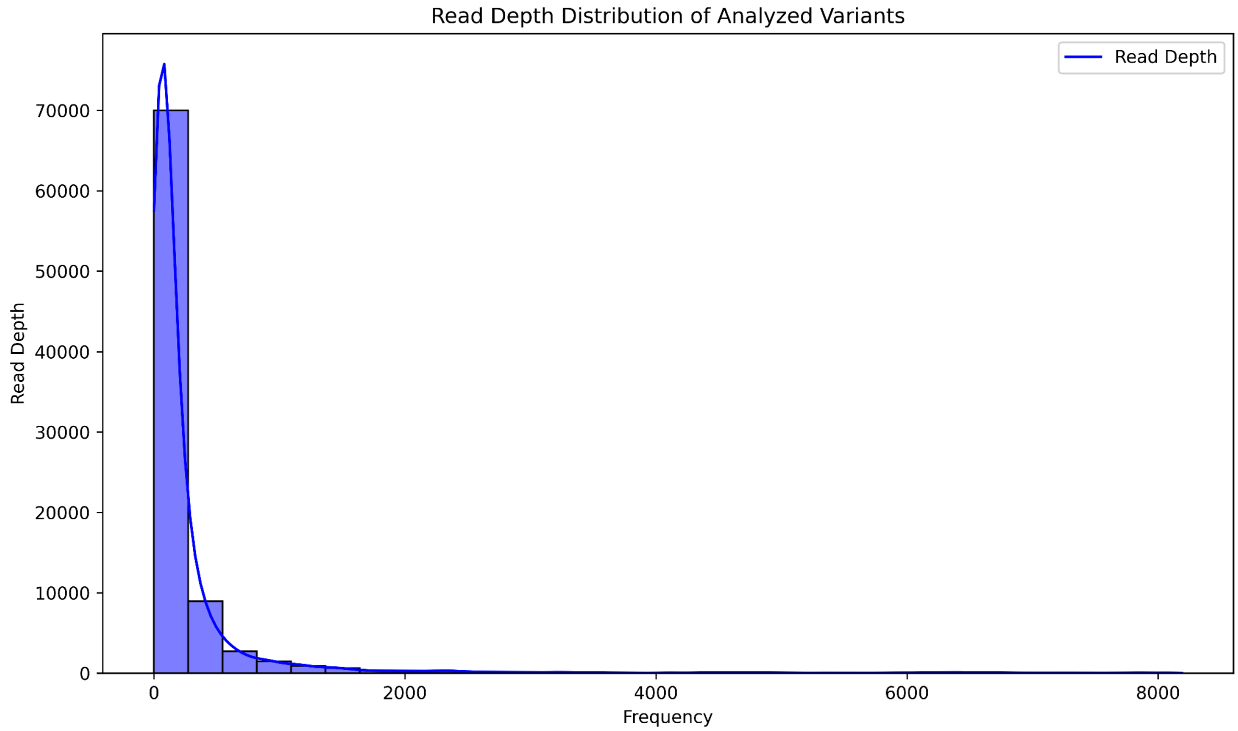

Supplementary Figure 2 - Distribution of Read Counts for Heterozygous Loci : The distribution of read depth for each of the loci used in this study’s analysis
