# Supplementary material for: A comprehensive allele specific expression resource for the equine transcriptome
Source: BMC Genomics. 2025 Jan 30;26:88. doi: 10.1186/s12864-025-11240-6 (PMC11780778; doi:10.1186/s12864-025-11240-6)
Supplement: Supplementary file 6 — Additional file 6: Supplementary Figure 3. Distribution of aeFC for all allele comparisons in this study. [file 12864_2025_11240_MOESM6_ESM.pdf]

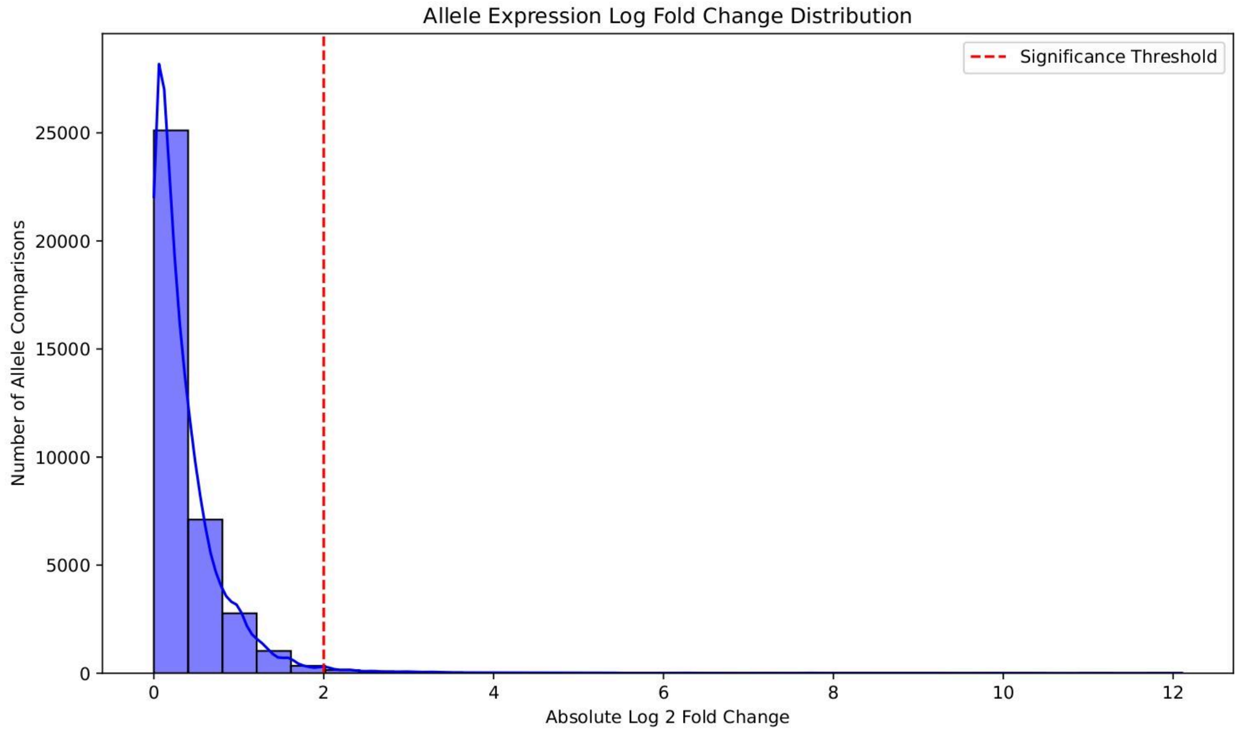

Supplementary Figure 3 - Distribution of Allele Expression Fold Change for All Allele Comparisons : The distribution of the allele expression fold changes for all of the loci analyzed in this study. The red dotted line represents the Allele expression fold change threshold for declaring a haplotype as potentially having a significant preference for being transcribed.
