# Supplementary material for: A comprehensive allele specific expression resource for the equine transcriptome
Source: BMC Genomics. 2025 Jan 30;26:88. doi: 10.1186/s12864-025-11240-6 (PMC11780778; doi:10.1186/s12864-025-11240-6)
Supplement: Supplementary file 7 — Additional file 7: Supplementary Figure 4. Frequency of ASE comparisons verified in our validation set. [file 12864_2025_11240_MOESM7_ESM.pdf]

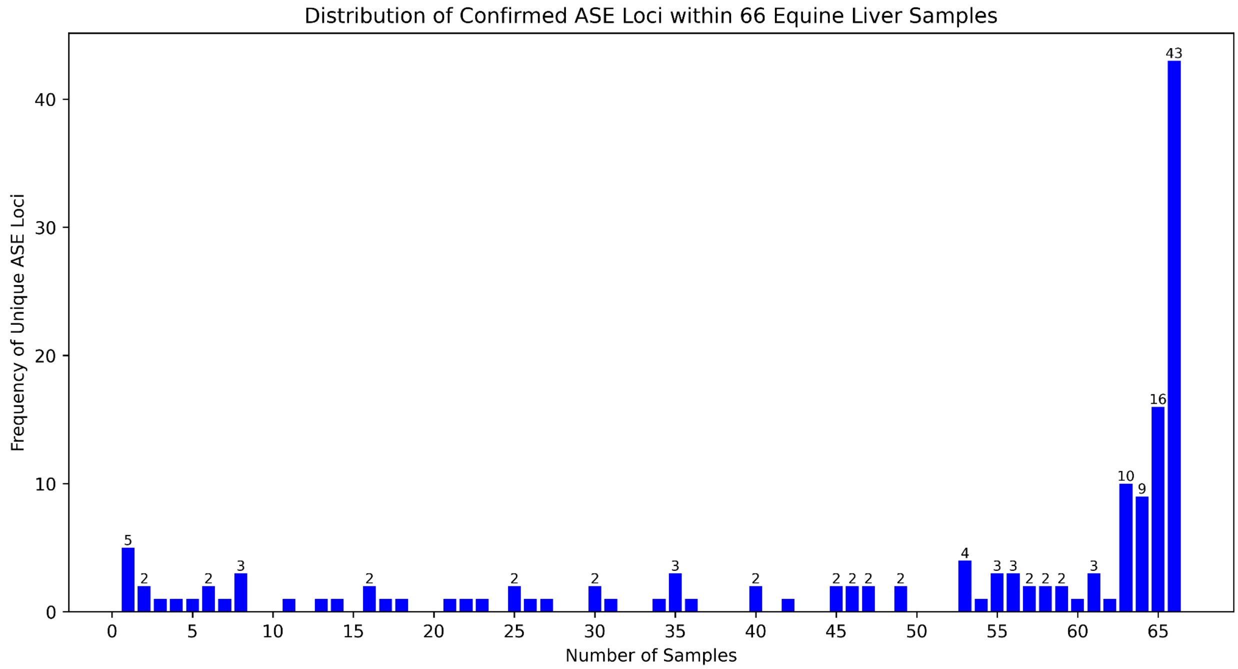

Supplementary Figure 4 - Frequency of ASE Comparisons Verified in the Validation Cohort : This plot shows the frequency that identified ASE loci were confirmed in our validation set. For example, 43 haplotypes were confirmed to exhibit ASE in all of our liver samples in the validation cohort.
