# Supplementary material for: A comprehensive allele specific expression resource for the equine transcriptome
Source: BMC Genomics. 2025 Jan 30;26:88. doi: 10.1186/s12864-025-11240-6 (PMC11780778; doi:10.1186/s12864-025-11240-6)
Supplement: Supplementary file 8 — Additional file 8: Supplementary Figure 5. Box and Violin Plots of the log fold change distribution of identified ASE loci across all breeds in our validation cohort. QH = Quarter Horse. [file 12864_2025_11240_MOESM8_ESM.pdf]

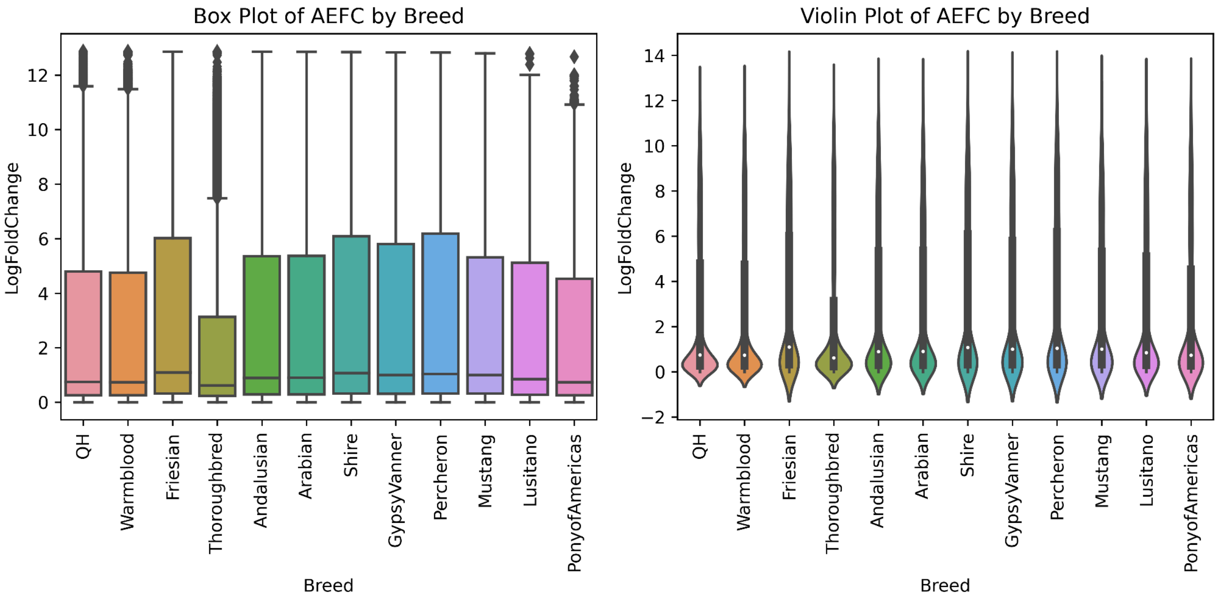

Supplementary Figure 5 - Log Fold Change Distribution of Identified ASE Loci Across All Breeds in the Validation Cohort : Box and Violin Plots of the log fold change distribution of identified ASE loci across all breeds in our validation cohort. QH = Quarter Horse
