# Supplementary material for: A comprehensive allele specific expression resource for the equine transcriptome
Source: BMC Genomics. 2025 Jan 30;26:88. doi: 10.1186/s12864-025-11240-6 (PMC11780778; doi:10.1186/s12864-025-11240-6)
Supplement: Supplementary file 10 — Additional file 10: Supplementary Figure 7. Pictures of a few validated ASE loci in Integrative; gene AHSG locus 3:27,077,158 (6), gene APOE locus 3:15,714,449 (7), gene CLU locus 3:56,553,608 (8). [file 12864_2025_11240_MOESM10_ESM.pdf]

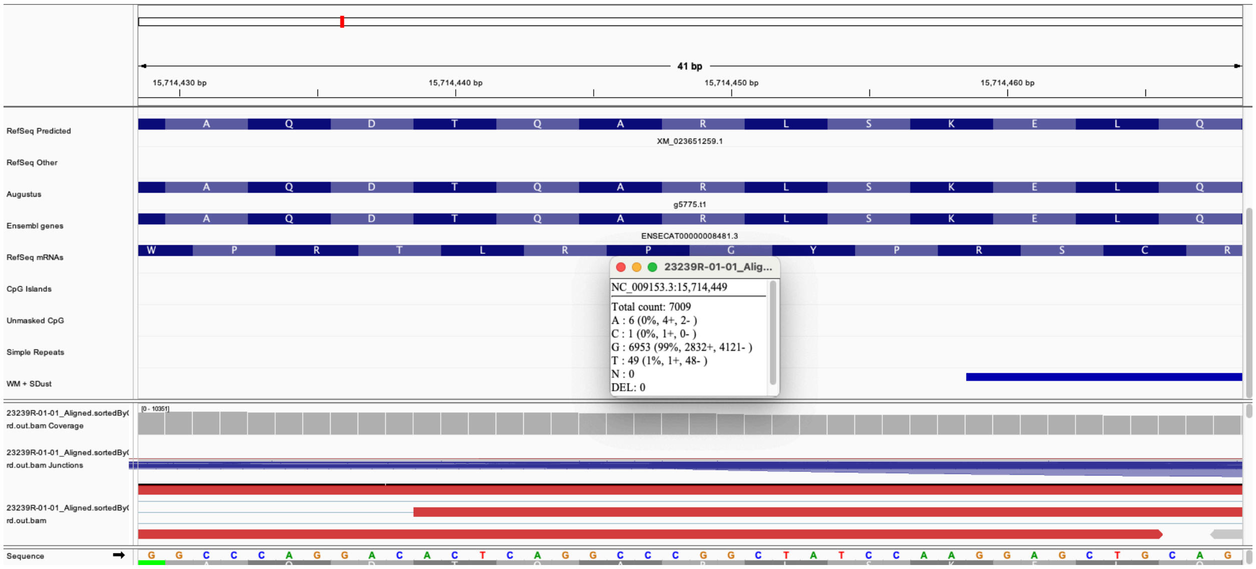

Supplementary Figure 7 - Validated ASE Locus in APOE Gene : Integrated Genome Viewer (IGV) screenshot showing the nucleotide counts of an ASE locus discovered in our original dataset, and confirmed in the validation set. This screen shot shows the results of this loci in one of the validation horses, and lies within the APOE gene.
